# A deep learning knowledge distillation framework using knee MRI and arthroscopy data for meniscus tear detection

**DOI:** 10.3389/fbioe.2023.1326706

**Published:** 2024-01-15

**Authors:** Mengjie Ying, Yufan Wang, Kai Yang, Haoyuan Wang, Xudong Liu

**Affiliations:** ^1^ Department of Orthopedics, Shanghai Sixth People’s Hospital Affiliated to Shanghai Jiao Tong University School of Medicine, Shanghai, China; ^2^ Engineering Research Center for Digital Medicine of the Ministry of Education, Shanghai, China; ^3^ School of Biomedical Engineering and Med-X Research Institute, Shanghai Jiao Tong University, Shanghai, China; ^4^ Department of Radiology, Shanghai Sixth People’s Hospital Affiliated to Shanghai Jiao Tong University School of Medicine, Shanghai, China

**Keywords:** knee joint, meniscal lesions, artificial intelligence, deep learning, computer-assisted diagnosis, magnetic resonance imaging, arthroscopy

## Abstract

**Purpose:** To construct a deep learning knowledge distillation framework exploring the utilization of MRI alone or combing with distilled Arthroscopy information for meniscus tear detection.

**Methods:** A database of 199 paired knee Arthroscopy-MRI exams was used to develop a multimodal teacher network and an MRI-based student network, which used residual neural networks architectures. A knowledge distillation framework comprising the multimodal teacher network *T* and the monomodal student network *S* was proposed. We optimized the loss functions of mean squared error (MSE) and cross-entropy (CE) to enable the student network *S* to learn arthroscopic information from the teacher network *T* through our deep learning knowledge distillation framework, ultimately resulting in a distilled student network *S* ^*T*^. A coronal proton density (PD)-weighted fat-suppressed MRI sequence was used in this study. Fivefold cross-validation was employed, and the accuracy, sensitivity, specificity, F1-score, receiver operating characteristic (ROC) curves and area under the receiver operating characteristic curve (AUC) were used to evaluate the medial and lateral meniscal tears detection performance of the models, including the undistilled student model *S*, the distilled student model *S* ^*T*^ and the teacher model *T*.

**Results:** The AUCs of the undistilled student model *S*, the distilled student model *S* ^*T*^, the teacher model *T* for medial meniscus (MM) tear detection and lateral meniscus (LM) tear detection are 0.773/0.672, 0.792/0.751 and 0.834/0.746, respectively. The distilled student model *S* ^*T*^ had higher AUCs than the undistilled model *S*. After undergoing knowledge distillation processing, the distilled student model demonstrated promising results, with accuracy (0.764/0.734), sensitivity (0.838/0.661), and F1-score (0.680/0.754) for both medial and lateral tear detection better than the undistilled one with accuracy (0.734/0.648), sensitivity (0.733/0.607), and F1-score (0.620/0.673).

**Conclusion:** Through the knowledge distillation framework, the student model *S* based on MRI benefited from the multimodal teacher model *T* and achieved an improved meniscus tear detection performance.

## 1 Introduction

The menisci are two fibrocartilaginous discs located between the femur and the tibia in each knee that work to stabilize the knee joint and distribute the load. Meniscus tears are common and severe since they can lead to articular cartilage degeneration with the risk of progression to osteoarthritis (Kopf et al., 2020; Li et al., 2020). Early diagnosis and treatment help in preventing osteoarthritis (Martel-Pelletier et al., 2016). Arthroscopy with high resolution is often considered the gold standard for diagnosis and can directly observe the internal tissue of the knee joint (Krakowski et al., 2019). Due to the high cost and invasive operation, knee arthroscopic data are difficult to obtain. Magnetic resonance imaging (MRI) is a noninvasive examination that provides cross-sectional information to detect meniscus tears and is more commonly utilized in the diagnosis and treatment of meniscus injuries. However, compared to the gold standard of arthroscopic examination, human identification of medial meniscus tears using MRI shows a sensitivity of 89% and specificity of 88%, while for lateral meniscus tears, the sensitivity and specificity are 78% and 95% (Phelan et al., 2016). Human identification of MRI images is limited by subjectivity, variability among interpreters, cognitive fatigue, processing constraints, experience sensitivity, and time pressures. Therefore, the efficiency of manually detecting knee joint MRI for diagnosing meniscus injury is still insufficient.

In recent years, deep learning has become a transformative tool across various fields. Its ability to automatically learn hierarchical representations from data has significantly impacted medical image analysis, offering unprecedented insights and facilitating the development of diagnostic methods. The application of deep learning in medical imaging has made significant progress, and convolutional neural networks have shown extraordinary abilities in tasks such as image segmentation, disease detection, and diagnostic decision support. The ability of deep neural networks to distinguish complex patterns in medical images greatly promotes our understanding and interpretation of complex pathology and diseases. Deep learning has gained much attention and could utilize medical imaging to diagnose the knee joint abnormalities (Bien et al., 2018; Pedoia et al., 2019; Rizk et al., 2021). Multimodal learning is an approach that combines multiple sources of data to provide more information and better performance (Kong et al., 2022). Integrating morphological information from knee MRI exams and arthroscopy provides a possibility for supplying more comprehensive information and detecting more detailed meniscus injuries. The significance lies in leveraging the complementary information from both MRI and arthroscopic imaging modalities to construct a deep learning network, enhancing diagnostic accuracy and robustness in joint pathology assessment. Considering that arthroscopic images are commonly acquired during surgery, which is after disease diagnosis, knee arthroscopic data can only be leveraged as missing modality (Chevalier et al., 2018; Shaikh et al., 2020; Ganaie and Tanveer, 2022) input for training but not for testing in deep learning (Tung and Mori, 2019).

Meanwhile, knowledge distillation has become a focal point in the evolution of deep learning models. Originally conceived as a technique for model compression, knowledge distillation has transcended its initial purpose. It serves not only as a means to reduce model complexity but also as a mechanism for transferring the acquired knowledge from a teacher model to a more lightweight student model. This pedagogical approach not only facilitates the deployment of models in resource-constrained environments but also contributes to model generalization and interpretability. Knowledge distillation-based methods can be utilized to address the absence of modalities in medical image analysis (Chen et al., 2022). However, whether knowledge distillation can be used to teach missing arthroscopic information to mono-modality MRI model to improve network performance remains to be verified.

Thus, the purpose of this study was to investigate whether the mono-modal student model *S* with only MRI input could learn the knowledge of knee arthroscopy from the multimodal teacher model *T* through a knowledge distillation learning framework and improve the performance in meniscus tear detection.

## 2 Materials and methods

### 2.1 Study population

**FIGURE 1:**
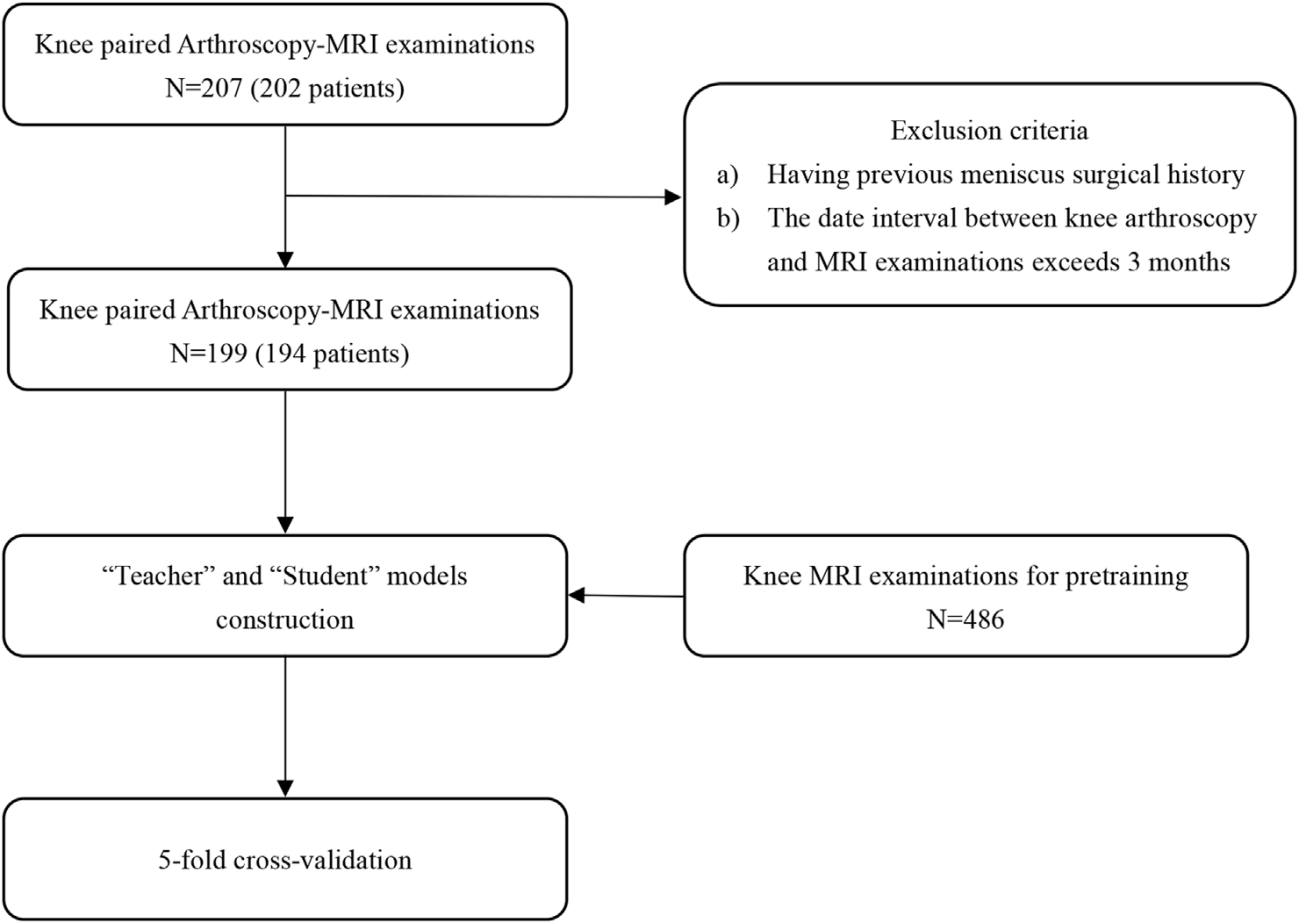
Schematic diagram of knee MRI and arthroscopy data utilization process.

This retrospective study included a total of 202 patients who had knee MRI examinations and their paired arthroscopic images at Shanghai Sixth People’s Hospital Affiliated with Shanghai Jiao Tong University School of Medicine between January 2021 and December 2022. Patients who had a previous knee surgery history or an interval between the MRI examination and subsequent surgery of more than 3 months were excluded (8 patients). The included population consisted of 87 (44.8%) men and 107 (55.2%) women with a mean age of 40.5 years and a standard deviation (SD) of 15.3 years. Five of them had surgical images and MRI exams on both knees (194 patients, 199 cases). The data composition and utilization of our research are shown in Figure 1. There were 169 injury cases with 60 (30.2%) medial meniscal tears and 125 (62.8%) lateral meniscal tears in the paired knee Arthroscopy-MRI dataset. The report of the knee surgery served as the standard of reference of this study. Study population information from our databases is detailed in Table 1.

**TABLE 1:** Summary statistics of the study population from our datasets.

Statistic	MRI pretraining dataset	Arthroscopy-MRI dataset	
Number of exams	486	199	
Training set	388	159/160	
Testing set	98	40/39	
Number of patients	486	194	
Number of female patients (%)	—	107 (55.2)	
Age, mean (SD)	44.4 (16.1)	40.5 (15.3)	
Number with medial meniscal tear (%)	151 (31.1)	60 (30.2)	
Number with lateral meniscal tear (%)	84 (17.3)	125 (62.8)	

### 2.2 Image data acquisition and preprocessing

All MRI scans were collected on a 3.0-T MRI Scanner (Achieva; Philips Healthcare, Netherlands). Our study used the coronal proton density (PD)-weighted fat-suppression sequence with the following scanning settings: repetition time, 2,100 ms; echo time, 38.4 ms and slice thickness, 3.2 mm. The MRI images were labelled medial intact, medial injury, lateral intact or lateral injury, and the region of interest (ROI) was cropped based on the manual segmentation results.

The knee arthroscopic surgical videos of the study, saved in 720*576 resolution with MPG format, were collected by a 660HD Image Management System (Smith and Nephew, United States) using an HD Autoclavable Camera System (Smith and Nephew, United States). The surgical videos were then converted into 720*576 resolution images with JPEG format at 10 frames per second. The arthroscopic images containing the meniscus were selected as input data for training of the teacher model *T*.

The cropped MR images and the selected arthroscopic images, resized to unified sizes separately, were applied with Min-Max normalization to eliminate the impact of data dimensionality on modelling and promote algorithm convergence (Jain et al., 2005). Considering the robustness of the models and the requirement of a large amount of data in deep learning, a data augmentation strategy was applied with random rotation, random contrast adjustment and random addition of Gaussian noise to the images.

### 2.3 Knee MRI network pretraining dataset

Pre-training is a common strategy in machine learning and deep learning, where a model is initially trained on a broad dataset to acquire general knowledge and patterns. The model is first trained on a diverse dataset to learn general features and patterns. Once this pre-training phase is completed, the model can be fine-tuned on a smaller, task-specific dataset to improve its performance on the target task. To mitigate the risk of overfitting and address data scarcity, we pre-trained a meniscus injury detection network. The knee MRI network pretraining dataset contained 486 cases of patients who had knee MRI examinations that were collected and deidentified from the same institution as previously described. This dataset was used mainly to develop a pretrained residual neural network for knee MRI feature extraction and further transfer learning. The pretrained weights were preserved for the training of the student model and the teacher model’s MRI network.

### 2.4 Development of the algorithm framework

We developed a deep learning knowledge distillation framework for medial and lateral meniscus tear detection, where the learning performance of the distilled student model *S* ^*T*^ relied on distillation strategy, as well as the teacher-student architecture and knowledge type in a knowledge distillation framework (Gou et al., 2021).

**FIGURE 2:**
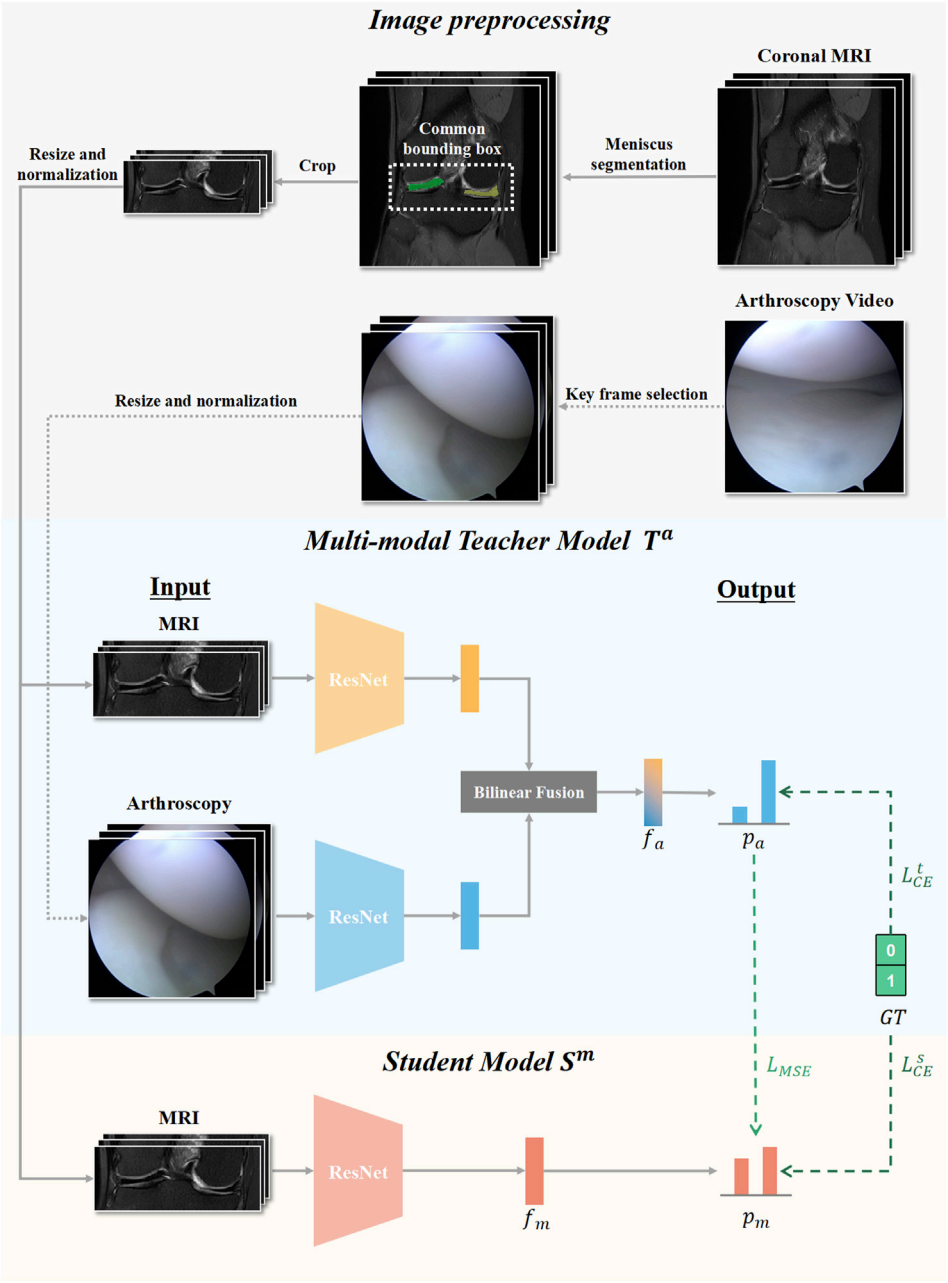
Flow chart of our knowledge distillation framework. Knee MRI examinations and Arthroscopic images were preprocessed and input for developing the multimodal teacher model T^a^, and MRI examinations were additionally utilized to develop the mono-modal student model S^m^. The student modal S^m^ distilled knowledge from the teacher modal T^a^ by optimizing the loss of mean square error L_MSE_ and the loss of cross-entropy L^s^_CE_.

In this study, our knowledge distillation method was performed offline. The teacher model *T* output the logits and produced the soft targets to guide the training of the student model *S* during distillation. In this way, even without arthroscopic image input, the student model *S* that only accessed MR images could still learn valuable information from the teacher model *T*, thus improving the performance of meniscus tear detection. Arthroscopic knee images as missing modality could be transferred from the teacher model into the student model through knowledge distillation. Our algorithm framework flowchart is presented in Figure 2.

All network architectures in this study used ResNet-18 as the backbone, which is a representative deep convolutional neural network that includes convolutional layers, pooling layers, fully connected layers, and residual connections (He et al., 2016). ResNet-18 excelled with a balanced trade-off between model complexity and efficiency, offering advantages in faster training, lower memory requirements, and well-suited performance for various deep learning tasks. To demonstrate the results in a more intuitive and universally applicable way, we chose to use ResNet-18 to construct a knowledge extraction framework. We also constructed knowledge distillation frameworks that composed of other residual neural network architectures, and the results are presented in Supplementary Table S1. The student model *S* was based on the pretrained residual neural network with only MR image input. The teacher model *T* consisted of two feature extractors with the same architecture based on residual network, one for MR image analysis and another for arthroscopic feature extraction. To utilize the valuable multimodal information, in the construction of the teacher model, we utilized two individual streams to process data of each modality and then integrated the multimodal knowledge at the intermediate feature level by bilinear fusion, which was widely used to combine imaging features from two different sources (Peng et al., 2020; Ni et al., 2022). During training, the Adam optimizer was utilized with an initial learning rate of 0.0001 and a weight decay of 0.0001 to control overfitting and improve generalization performance. An early stopping strategy was adopted, in which training was stopped if the loss metric did not improve within 100 iterations.

We adopted the response-based knowledge distillation method, which transferred the knowledge captured by soft targets of the teacher model (Hinton et al., 2015). To adaptively absorb valuable knowledge from the teacher model, the algorithm framework employed the optimization of two loss functions as supervision: the mean squared error (MSE) loss and the cross-entropy (CE) loss.

Our algorithm framework employed 5-fold cross-validation to assess the detection performance of the models, which helped in understanding the models’ ability to correctly classify instances and the overall effectiveness. Heatmaps for the distilled student model were generated to show the highlighted regions within images.

### 2.5 Implementation details

Our training process was performed in a Linux environment on an NVIDIA A100 SXM4 graphics processing unit (GPU) with 80 GB random access memory (RAM). The whole knowledge distillation framework was implemented with Python 3.9.16 and Torch 1.8.1 + cu111.

### 2.6 Statistical analysis

The following statistical analyses were performed by using SPSS (Version 26, IBM Cooperation, United States). For continuous values, data are shown as the mean with standard deviation. By employing 5-fold cross-validation, the paired Arthroscopy-MRI dataset was randomly shuffled and split into five subsets in which four of the subsets contained 40 cases and the remaining subset contained 39 cases. In each iteration, our models selected a fold as the validation set and the remaining four folds together as the training set. Each fold serves as the validation set one time, and our models were evaluated across five iterations to obtain an overall assessment. We compared the performance in medial and lateral meniscus tear detection of the undistilled student model *S*, the distilled student model *S* ^*T*^ and the teacher model *T* with the metrics of accuracy, sensitivity, specificity, F1-score and area under the receiver operating characteristic curve (AUC). We chose the threshold that maximized the Youden index for analysis. The performance of the above three models was also evaluated with receiver operating characteristic (ROC) analysis.

## 3 Results

For medial meniscus (MM) tear detection and lateral meniscus (LM) tear detection, the student model *S* achieved AUCs of 0.773 and 0.672, respectively. The teacher model *T* achieved AUCs of 0.834 in MM and 0.746 in LM. The student model after knowledge distillation *S* ^*T*^ achieved an AUC of 0.792 in MM and 0.751 in LM, which were higher than the undistilled student model *S*. The results of our three models for each fold with the overall AUC in medial and lateral meniscus tear detection are presented in Table 2.

**TABLE 2:** Overall AUC results of the three models: student model, distilled student (teacher-student) model and teacher model on each fold in meniscus tear detection.

Overall AUC	Fold1	Fold2	Fold3	Fold4	Fold5	Averaged
Student	0.729	0.695	0.727	0.722	0.745	0.724
Teacher-Student	0.766	0.749	0.781	0.767	0.789	0.770
Teacher	0.793	0.755	0.807	0.796	0.801	0.790

**FIGURE 3:**
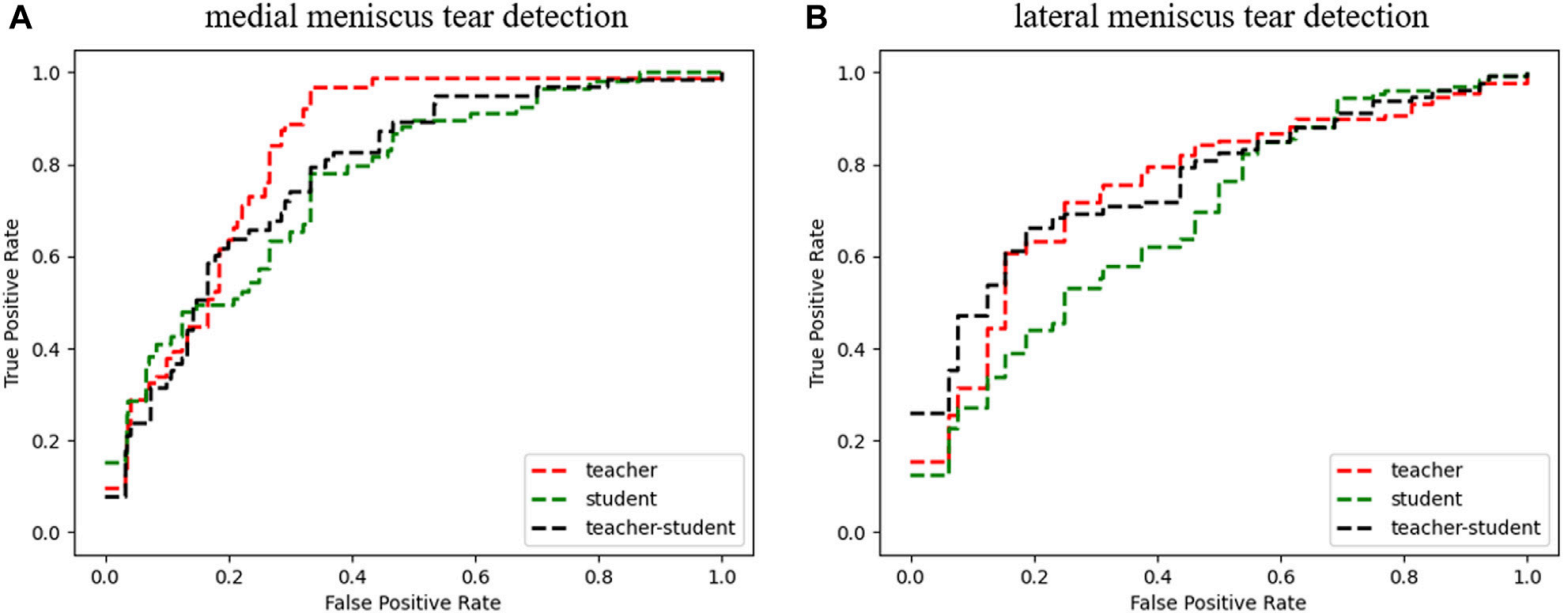
Receiver operating characteristic curve of the three models in medial **(A)** and lateral **(B)** meniscus tear detection.

The threshold that optimized the Youden index was chosen. For the student model *S*, the accuracy, sensitivity, specificity, F1-score and AUC values in medial/lateral meniscal tear detection were 0.734/0.648, 0.733/0.607, 0.735/0.741, 0.620/0.673 and 0.773/0.672, respectively. For the teacher model *T*, the accuracy, sensitivity, specificity, F1-score and AUC values in medial/lateral meniscal tear detection were 0.779/0.744, 0.967/0.700, 0.697/0.826, 0.721/0.766 and 0.834/0.746, respectively. The performance of the teacher model *T* was superior to that of the student model *S*, except for the specificity in detecting medial meniscus tears. For the distilled student model *S* ^*T*^, the accuracy, sensitivity, specificity, F1-score and AUC values in medial/lateral meniscal tear detection were 0.764/0.734, 0.838/0.661, 0.722/0.851, 0.680/0.754 and 0.792/0.751, respectively. Thus, the performance of the student model after knowledge distillation *S* ^*T*^ was close to that of the teacher model *T* and better than the undistilled student model *S*. The performance summary metrics of our student model, distilled student model and teacher model can be found in Table 3. The receiver operating characteristic curves (ROCs) of our three models are shown in Figure 3.

**TABLE 3:** Diagnostic performance of our three models to detect medial and lateral meniscus tears.

	Accuracy	Sensitivity	Specificity	F1-score	AUC
Student					
Medial	0.734	0.733	0.735	0.620	0.773
Lateral	0.648	0.607	0.741	0.673	0.672
Teacher-student					
Medial	**0.764**	**0.838**	0.722	**0.680**	**0.792**
Lateral	**0.734**	**0.661**	**0.851**	**0.754**	**0.751**
Teacher					
Medial	0.779	0.967	0.697	0.721	0.834
Lateral	0.744	0.700	0.826	0.766	0.746

The bold results represent the metrics of the distilled student network, which are better than those of the undistilled student network.

Heatmaps were generated to better discern which areas of the image were the most focused on. The generated highlight region focused on the medial and lateral meniscus of the cropped MR images. (Supplementary Figure S1).

## 4 Discussion

These results indicated that the distilled student model *S* ^*T*^, which learned arthroscopic knowledge from the teacher model *T*, outperformed the undistilled student model *S*. In this paper, we present the study to integrate knee arthroscopic knowledge into MRI diagnostic model for more accurate meniscus tear detection.

Most studies for knee meniscus tear detection in deep learning were formulated using MR images. Bien et al. (2018) developed MRNet, a convolutional neural network, based on three MRI series for detecting meniscus tears and anterior cruciate ligament (ACL) injuries. Key et al. (2022) created three different MRI corpora for automatic meniscus tear diagnosis model development. While multimodal data can provide comprehensive and complementary information for disease diagnosis and treatment planning (Fan et al., 2022; Gao et al., 2022). MRI is a non-invasive technique providing excellent soft tissue contrast and allowing for multi-planar imaging, while it has limitations in detecting degenerative tears or small tears that are not clear on the images. Arthroscopy is considered the gold standard for diagnosing certain joint abnormalities, providing high-resolution images, but it is an invasive, expensive and time-consuming procedure. Therefore, arthroscopy data are rarely collected for preoperative diagnosis studies, and studies based on arthroscopy have mostly focused on integrating arthroscopy with other radiological examinations to enhance intraoperative navigation and improve surgical outcomes (Kotheeranurak et al., 2019; Shigi et al., 2019; Chen et al., 2021). Both detection methods have their own advantages and can complement each other. There is currently no research using knee MR images and corresponding arthroscopic images as input for meniscus tear detection model development. Thus, we propose to leverage the advantages of the knee MRI and arthroscopy data to conduct multimodal fusion learning for enhanced meniscus injury detection.

Multimodal learning typically involves leveraging multiple modalities during both training and inference stages to achieve optimal performance (Venugopalan et al., 2021; Zhang et al., 2021). In many scenarios, multiple modalities of high-quality training data can be well prepared, but during real-world evaluation, only one modality can be accessible (Wang et al., 2022; Xiong et al., 2023). This is a common challenge in that decisions and evaluations often need to be made based on limited information. A knowledge distillation framework with a ‘Teacher-Student’ architecture is proposed, in which the missing modality information available in the training data can be transferred from a multimodal teacher model to a mono-modal student model. Guan et al. (2021) proposed a multi-instance distillation programme that distilled the knowledge learned from multimodal data into an MRI-based model to address the task of mild cognitive impairment conversion prediction. Gao et al. (2020) employed knowledge distillation to support network learning with the target modality alone for vessel border detection. To our knowledge, we proposed the first work that distilled knee arthroscopic information to an MRI-based student model. By leveraging the arthroscopic information during training and distilling its knowledge to the student model, our study enabled the MRI modality to benefit from the additional information contained in the knee arthroscopy modality.

Several limitations of this research should be acknowledged. First, all patients have undergone arthroscopy confirmation, so our single-center dataset has a small sample size, which may lead to bias. Due to the small size of the dataset, it may not fully reflect the diversity and complexity of the entire patient population. Such training datasets may not represent the characteristics of different medical centers, regions, or populations well, leading to bias in the trained deep learning models when generalized to other environments or populations. Second, arthroscopic images have characteristics such as low contrast, interference of fat droplets, and intra-articular tissue reflection caused by light sources, which results in poor feature extraction performance. Further research is needed on the network for image processing and feature extraction of knee arthroscopic images to address the above issues. Third, due to the small sample size, the model classification is only divided into “intact” or “injury” categories. According to the results of knee arthroscopy surgery, we only define the level III (linear high signal shadow reaches the upper or lower surfaces of the meniscus in MRI) meniscus injury as “injury,” but our models are difficult to distinguish the level I (Clustered low brightness shadow in MRI) and level II (linear high signal shadow, but not reaches the upper or lower surfaces of the meniscus in MRI) meniscus injury from the level III meniscus injury, resulting in only two classification labels being presented in the final classification result. Last, the clinical significance of the meniscus injury diagnosis model constructed using the knowledge distillation framework needs further research. Multiple radiologists with different levels of experience needed to be invited to diagnosis knee meniscus tears with or without the assistance of artificial intelligence models, and compare the results for validating if the models add value in real clinical practice.

## 5 Conclusion

It was demonstrated that the distilled student model *S* ^*T*^ achieved more competitive performance compared with the student model *S* after learning arthroscopic information from the teacher model *T* through knowledge distillation. Further studies using larger datasets and exploring various knowledge distillation frameworks are needed to validate the effectiveness of knowledge distillation and consolidate our findings.

## Data Availability

The raw data supporting the conclusion of this article will be made available by the authors, without undue reservation.
