# Supplementary material for: A deep learning knowledge distillation framework using knee MRI and arthroscopy data for meniscus tear detection
Source: Front Bioeng Biotechnol. 2024 Jan 15;11:1326706. doi: 10.3389/fbioe.2023.1326706 (PMC10825958; doi:10.3389/fbioe.2023.1326706)
Supplement: Supplementary file 1 [file Table1.docx]

Supplementary Material

We experimented with different residual network architectures and constructed corresponding knowledge extraction frameworks under other unchanged conditions. We have compared various indicators for the undistilled student network *S* and the distilled student network *S^T^*.

**Supplementary Table 1** Overall metrics for knowledge extraction frameworks constructed by different residual network architectures.

| **Overall metrics** | |  | **Accuracy** | **Sensitivity** | **Specificity** | **F1-score** | **AUC** |
| --- | --- | --- | --- | --- | --- | --- | --- |
| **Resnet34** | *S* | | 0.652 | 0.617 | 0.704 | 0.677 | 0.624 |
|  | *S^T^* | | **0.679** | 0.611 | **0.745** | 0.657 | **0.653** |
| **Resnet50** | *S* | | 0.626 | 0.593 | 0.696 | 0.615 | 0.588 |
|  | *S^T^* | | **0.674** | **0.719** | 0.672 | **0.689** | **0.666** |
| **Resnet101** | *S* | | 0.616 | 0.639 | 0.657 | 0.624 | 0.572 |
|  | *S^T^* | | **0.657** | **0.664** | **0.675** | **0.661** | **0.671** |
| **Resnet152** | *S* | | 0.636 | 0.665 | 0.668 | 0.646 | 0.596 |
|  | *S^T^* | | **0.657** | **0.694** | 0.663 | **0.669** | **0.646** |

Bold numbers represent better performance of the distilled student network *S^T^* compared to the undistilled student network *S* in different frameworks.
