# Supplementary material for: A deep learning knowledge distillation framework using knee MRI and arthroscopy data for meniscus tear detection
Source: Front Bioeng Biotechnol. 2024 Jan 15;11:1326706. doi: 10.3389/fbioe.2023.1326706 (PMC10825958; doi:10.3389/fbioe.2023.1326706)

Supplementary Material

Heatmaps were generated to better discern which areas of the image were the most focused on. The generated highlight region focused on the medial and lateral meniscus of the cropped MR images.

Supplementary Figure 1 Heat maps for meniscus damage detection.

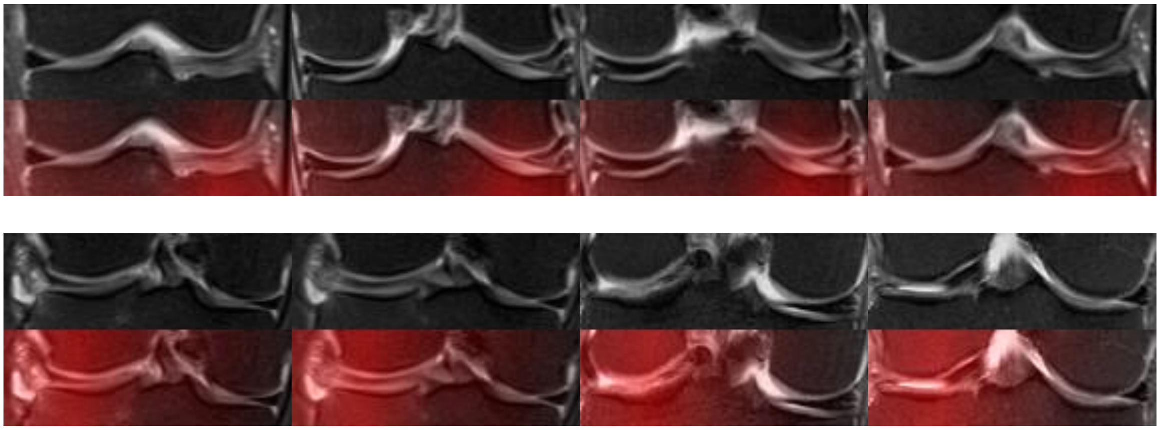

Supplement: Supplementary file 2 [file Image1.pdf]
